# Access to myocardial revascularization procedures: Closing the gap with time?

**DOI:** 10.1186/1471-2458-6-60

**Published:** 2006-03-08

**Authors:** Alain Vanasse, Théophile Niyonsenga, Josiane Courteau, Abbas Hemiari

**Affiliations:** 1 Family Medicine Department, Faculty of Medicine, *Université de Sherbrooke*, 3001, 12^th ^Avenue North, Sherbrooke (QC), J1H 5N4, Canada; 2 PRIMUS Group, Clinical Research Center, Sherbrooke University Hospital, Sherbrooke (QC), Canada; 3 Epidemiology & Biostatistics, Stempel School of Public Health, Florida International University (FIU), USA

## Abstract

**Background:**

Early access to revascularization procedures is known to be related to a more favorable outcome in myocardial infarction (MI) patients, but access to specialized care varies widely amongst the population. We aim to test if the early gap found in the revascularization rates, according to distance between patients' location and the closest specialized cardiology center (SCC), remains on a long term basis.

**Methods:**

We conducted a population-based cohort study using data from the Quebec's hospital discharge register (MED-ECHO). The study population includes all patients 25 years and older living in the province of Quebec, who were hospitalized for a MI in 1999 with a follow up time of one year after the index hospitalization. The main variable is revascularization (percutaneous transluminal coronary angioplasty or a coronary artery bypass graft). The population is divided in four groups depending how close they are from a SCC (<32 km, 32–64 km, 64–105 km and ≥105 km). Revascularization rates are adjusted for age and sex.

**Results:**

The study population includes 11,802 individuals, 66% are men. The one-year incidence rate of MI is 244 individuals per 100,000 inhabitants. At index hospitalization, a significant gap is found between patients living close (< 32 km) to a SCC and patients living farther (≥32 km). During the first year, a gap reduction can be observed but only for patients living at an intermediate distance from the specialized center (64–105 km).

**Conclusion:**

The gap observed in revascularization rates at the index hospitalization for MI is in favour of patients living closer (< 32 km) to a SCC. This gap remains unchanged over the first year after an MI except for patients living between 64 and 105 km, where a closing of the gap can be noticed.

## Background

In Canada, cardiovascular events represent a major health burden similar to what is observed in other developed countries. Myocardial infarction (MI) accounts for a large percentage of these events [1]. Although practice guidelines regarding MI management have been widely published [2,3], regional disparities in managed cares and outcomes are still observed in Canada [4-15], as well as in other countries [16-23].

Early percutaneous transluminal coronary angioplasty (PTCA) has been given much greater priority in recent years [3]. According to Health Canada, the number of PTCA done in Canada increased of 36% between 1994/95 and 2000/01 [24]. Moreover, patients suffering from a MI, who received an invasive cardiac procedure such as coronary angiography or revascularization during the first hospitalization, were less likely to be readmitted later for cardiac reasons [25]. Invasive cardiac procedures require technical facilities and a professional expertise available only in specialized cardiology centers, logically privileging people living closer. In fact, both the availability of these services in the hospital initially admitting the patient and the geographical proximity to these services are factors determining their use [5,26]. Thus patients from rural areas are disadvantaged as they are less likely to get a diagnostic and cardiac procedures [27], or must wait longer to access these procedures [28].

To our knowledge, only few studies have been published reporting results on the gap in revascularization rates according to the distance of patient's location and specialized cardiology centers (SCC). Amongst these, Scott et al [29] analyzed access to potential intravenous thrombolysis for stroke using crow-flight distances of 32, 64 and 105 km around Canadian hospitals. Vanasse et al [4] studied management and outcomes of acute coronary syndrome stratified by distance to SCC. This latter study showed that distances to SCC have an impact on the likelihood of receiving an invasive cardiac procedure at index hospitalization for patients suffering from an acute coronary syndrome. However, no one has studied the evolution of the gap over a one year period.

We hypothesize that MI patients living within a short distance from SCC will have a greater proportion of revascularization procedures compared to patients living farther, and that the gap will decrease with time, as patients living farther will have access to SCC on an elective basis in the year following their first hospitalization.

The aim of this paper is to test if the early gap observed in the revascularization rates according to distance between the patients' location and the closest SCC remains on a long term basis. The first objective of this study is to describe and compare the revascularization rates (PTCA or coronary artery bypass graft (CABG)) of patients with MI, according to distance categories (<32 km, 32–64 km, 64–105 km and ≥105 km). The second objective is to measure the evolution of the gap between groups over time (3, 6, 9, and 12 months).

## Methods

### Design

We conducted a population-based cohort study using secondary data analysis from the Quebec's hospital discharge register: « *Maintenance et Exploitation des Données pour l'Étude de la Clientèle Hospitalière (MED-ECHO) *». This register provides administrative data on all patients hospitalized in an acute care hospital in the province of Quebec. Studies confirming the accuracy of the administrative data concerning myocardial infarction have previously been published [30,31]. The follow-up period time is 12 months for every patient entered in the cohort.

### Studied population

The study population includes all patients 25 years and older living in the province of Quebec, who were hospitalized for a MI (code 410 of the IDC-9 International Disease Classification, 9^th ^revision) between January 1^st ^1999 and December 31^st ^1999. The "index hospitalization" is the first hospitalization during the study period. If the care management occurred over several contiguous hospitalizations involving hospital transfers, the index hospitalization refers to the entire episode. Patients with a MI in the year preceding the index hospitalization are excluded in order to include only new or stable MI cases. Patients from *Outaouais *are excluded because of its close proximity to a SCC located in Ontario, a contiguous province for which data is not available in the MED-ECHO register. Because of the very sparse population and a different care management pattern in the Northern Quebec region, patients from this region are excluded. Patients with an unknown postal code are also excluded.

The population is divided in four study groups. The geographic grouping of patients is based on the crow-flight distance between their living dwelling and the nearest SCC (<32 km, 32–64 km, 64–105 km and ≥105 km). We selected these cut points based on estimated travel times of 60, 90 and 120 minutes respectively to cover a distance of 32, 64 and 105 km [29,32]. The localization of the patient's area and the specialized cardiology center is defined by the geometrical centroid of their postal codes.

### Data sources

Patient-data were obtained from Quebec's hospital discharge register (MED-ECHO). Each patient was then spatially referenced by his/her postal code using data from DMTI Spatial [33]. The list of SCC was obtained from the Quebec tertiary cardiology network of the Quebec Ministry of Health and Social Services [34]. The geographic coordinate system used for the cartographic presentation was GCS North American 1983.

### Studied variables

Patients are considered to have undergone a revascularization procedure at index hospitalization if there is a mention of PTCA or CABG as coded in the Quebec's hospital discharge registers (Canadian Classification of Diagnostic, Therapeutic, and Surgical Procedures (CCP) beginning with 480 to 483). Revascularization rates are also established over specific time periods, namely at 3, 6, 9 and 12 months following the index hospitalization.

### Analyses

Descriptive analyses by distance categories from dwelling to nearest specialized cardiology center were done. We used the Pearson χ^2 ^test for comparisons between proportions. All revascularization rates are adjusted for age and sex. A cartographic representation of the adjusted rates is also presented using specific catchment areas of <32 km; 32–64 km; 64–105 km; and ≥105 km. Statistical analyses were done using SAS 9.1 [35] and cartographic representations were made using ArcGIS 9.0 [36].

### Ethical considerations

This project was approved by the Sherbrooke University Hospital Ethics Board and the *Commission d'accès à l'information du Québec*.

## Results

A total of 12,646 individuals were hospitalized for MI in Quebec between January 1^st ^1999 and December 31^th ^1999. Of those, some patients were excluded: 9 because they were less than 25 years old, 289 because they had a MI the year prior to index hospitalization, 544 because they were living in the sparsely populated northern region or in the *Outaouais *region, and 2 because there was an error in their code of residence. Therefore, the study population totalled 11,802 individuals, of which 66% (*n *= 7733) were men. The average age was 66.5 years (± 13.8). The one-year incidence rate was 244 individuals per 100,000 inhabitants.

**Figure 1:**
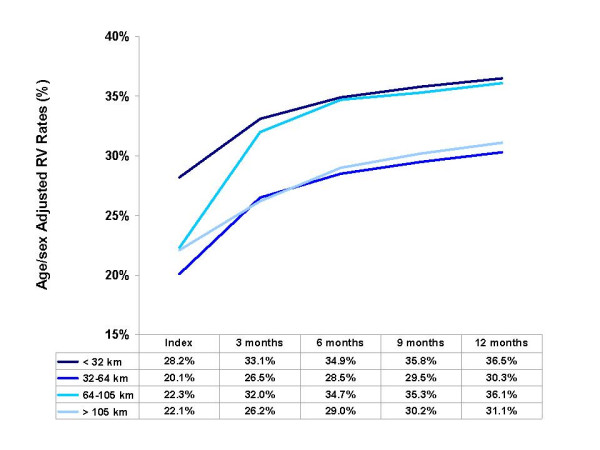
Age and sex adjusted revascularization (RV) rates at index hospitalization, 3, 6, 9 and 12 months after the index admission by distance from residence to nearest specialized cardiology center (n = 11,802).

**Figure 2:**
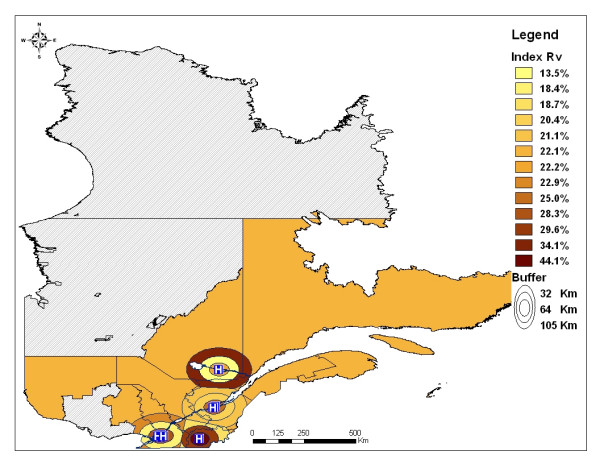
Age and sex adjusted revascularization (RV) rates at index hospitalization by distance from residence to specialized cardiology centers (n = 11,802).

**Figure 3:**
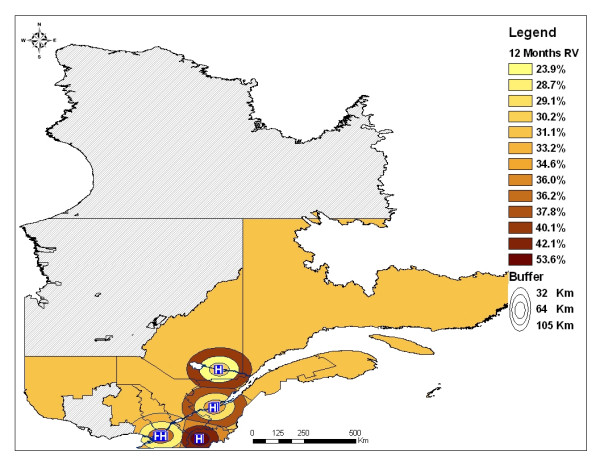
Age and sex adjusted revascularization (RV) rates at 12 months by distance from residence to specialized cardiology centers (n = 11,802).

The age and sex adjusted revascularization rates differed significantly (*p *< 0.0001) amongst groups and over time (Figure 1), varying from 20.1% to 28.2% at index hospitalization and from 30.3% to 36.5% one year after. Higher rates of revascularization are found in the patients' group closer to a SCC at all points in time. At index hospitalization, statistically significant gaps can be observed between the patients' group living close to a SCC (<32 km) and the other groups (p < 0.0001). During the first year, a reduction of the gap can be found but only for patients living at an intermediate distance from the specialized center (64–105 km). In fact, the gap in revascularization rates between patients living at less than 32 km and those living between 64 and 105 km from a SCC is not statistically significant after 3 months (p = 0.4812), but significant differences can be observed for the two other patients' groups (p < .0001), creating a halo pattern of low revascularization rates by patients living between 32 and 64 km from a SCC. This halo pattern is more easily demonstrated with the cartographic representations in Figure 2 and Figure 3.

## Discussion

Revascularization rates in the province of Quebec in 1999, as found in this study, are similar to others previously presented in Quebec [12]. The results showed above support the first part of our hypothesis. In fact, the gap in the revascularization rates between the four groups of patients at index hospitalization benefits patients living at less than 32 km from a SCC. A frequent explanation put forward in the literature to account for use of invasive cardiac procedures is accessibility to facilities performing such procedures [37].

The negative relationship between revascularization rates and distance to specialized centers has also been noticed in different countries [16,20,21,23] including Canada [5,7,11,13]. However the second part of our hypothesis is found to be partly true. Indeed, we can observe a complete closure of the gap for one of the groups living farther but the two others remain unchanged at one year. In fact, only the patients' group living within intermediate distances (64–105 km) to a SCC will close the gap over time. Strangely enough, the patients' group living between 32 and 64 km will experience the same lasting gap than the group of patient living farther than 105 km. This result cannot be attributed to a demographic difference between the four groups as age and sex adjusted rates have been presented. To our knowledge, it is the first time that the halo pattern revealed by the map has been described. Therefore, more comprehensive studies to fully understand this phenomenon are needed.

Piché et al argued that the relation between distance and access to care varies with the medical situation (severity, urgency, etc.) [38,39]. If we can logically hypothesize that patients in the 64–105 km radius of location may be easily transferred by their local physicians for non urgent revascularization than the patients living farther, no evident explanation can be offered for patients living in the 32–64 km radius.

In order to reduce bias in the comparison of each group, death rates were also measured at each point in time and were found to be not statistically different at the index hospitalization (p = 0.2628) and at one year (p = 0.1659) (data not presented). Bias can also result from an underestimation of deaths at index hospitalization in groups living farther and may possibly explain some of the differences. In fact, sicker people living in remote regions possibly die before even being admitted to the hospital. Increase in the time between onsets of symptoms to treatment (onset-to-door) has previously been reported in rural regions [40].

To better understand geographical disparities in healthcare management, more comprehensive exploration of socio-demographic variables' contribution is needed. Rurality is one of these variables that seems important to consider [41]. Indeed, rural populations differ from urban ones not only in geographical access to care [42] but also by their cultural and socioeconomic backgrounds. Other possible variables that can explain heterogeneity in MI management include social and material deprivation indices [43], medical care and professional attributes including academic affiliation and year of graduation for family physicians and cardiologists [15].

The use of administrative data has some limitations. Even though Quebec's hospital discharge register has been used for epidemiological studies on acute myocardial infarction [30,31], the follow-up of the hospital's care episode spread out over different care institutions requires an algorithm for which the accuracy needs to be further validated. Another limitation to take into account is the use of the crow-flight distance as estimated traveling time between the place of living and the care center [44,45]. The use of this proxy may not be valid for all regions in the province.

## Conclusion

According to distance categories, significant gaps were observed in revascularization rates after a MI at index hospitalization. The gaps remained significant during the first year after a MI, except for patients living at an intermediate distance (64–105 km) from the cardiology center. This halo pattern can be easily noticed on a thematic map.

## Competing interests

This project has benefited from an unrestricted grant by Merck Frosst Canada Ltd.

## Authors' contributions

AV, TN conceived the study, JC and TN performed the analyses. AV, TN and JC wrote the manuscript and AH produced the maps. All authors read and approved the final version of the manuscript.

## Pre-publication history

The pre-publication history for this paper can be accessed here:
